# Deciphering the Role of Fluorination in Dual‐Halogen Electrolytes for All‐Solid‐State Batteries: A Case Study of New Li_2_HfCl_6−x_F_x_ Solid Electrolytes

**DOI:** 10.1002/anie.202509209

**Published:** 2025-08-20

**Authors:** Lanting Qian, Yubo Wang, Jue Liu, Ivan Kochetkov, Ning Chen, Cameron Dean, Linda F. Nazar

**Affiliations:** ^1^ Department of Chemistry Waterloo Institute of Nanotechnology University of Waterloo Ontario N2L 3G1 Canada; ^2^ Department of Chemical Engineering University of Waterloo Ontario N2L 3G1 Canada; ^3^ Neutron Scattering Division, Oak Ridge National Laboratory Oak Ridge Tennessee 37822 USA; ^4^ Canadian Light Source 44 Innovation Blvd Saskatoon SK S7N 2V3 Canada

**Keywords:** Interphases, Lithium metal chloride electrolytes, Solid‐state batteries, Solid‐state electrolytes, ToF‐SIMS

## Abstract

Lithium metal chlorides are promising superionic conductors for all‐solid‐state batteries (SSBs) due to their favorable mechanical properties, high ionic conductivity, and good oxidative stability (up to >4.2 V versus Li/Li^+^). Nonetheless, chloride solid electrolytes (SEs) still undergo electrochemical degradation when paired with high‐voltage cathodes such as LiNi_0.85_Co_0.1_Mn_0.05_O_2_. A viable strategy to enhance the intrinsic electrochemical stability of chloride electrolytes is to partially substitute Cl with F. By leveraging complementary insights from neutron and X‐ray diffraction, X‐ray absorption spectroscopy, X‐ray photoelectron spectroscopy (XPS), time‐of‐flight secondary ion mass spectrometry (ToF‐SIMS), and electrochemical studies, we investigate the interplay between ionic and electronic conductivity, voltage stability, and overall battery performance of a family of new dual‐halogen SEs—Li_2_HfCl_6−x_F_x_. All‐solid‐state cells utilizing Li_2_HfCl_5.5_F_0.5_ as the electrolyte demonstrate much‐enhanced battery performance compared to Li_2_HfCl_6_. This improvement is mainly attributed to the formation of a kinetically stable LiF‐rich cathode electrolyte interphase (CEI), which inhibits detrimental reactions between the cathode and the SE, as revealed by ToF‐SIMS studies. The findings from this study are applicable to other dual‐halogen solid ionic conductors, offering valuable insights into the relationship between intrinsic electrochemical window (IEW), electronic and ionic conductivity, and battery performance in dual‐halogen solid‐state electrolytes.

## Graphical abstract

We synthesized a series of dual‐halogen solid electrolytes (SEs), based on a new *P* *nma* phase of Li_2_HfCl_6_. By systematically fluorinating it to various degrees, Li_2_HfCl_5.__5_F_0.5_ was identified as the optimal formulation. ASSBs assembled using it as the catholyte exhibited greatly enhanced electrochemical stability compared to those employing Li_2_HfCl_6_. We explicitly demonstrate that this is due to the formation of an in‐situ‐generated, LiF‐rich cathode‐electrolyte interphase, not enhanced anodic stability of the catholyte.

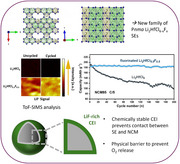

## Introduction

Solid‐state batteries (SSBs) have garnered strong interest as a next‐generation energy storage technology, owing to their potential for enhanced safety and higher energy density compared to current lithium‐ion batteries. Amongst the different types of solid electrolytes (SEs),^[^ ^1^, ^2^, ^3^, ^4^ ^]^ halide SEs are attractive due to their wide electrochemical window and high ionic conductivity. In particular, chloride SEs such as Li_3_InCl_6_,^[^ ^5^ ^]^ Li_3_YCl_6_,^[^ ^6^ ^]^ Li_3−3x_M_1+x_Cl_6_ (0.14 < *x* ≤ 0.5, M = Tb, Dy, Ho, Tm),^[^ ^7^ ^]^ Li_3_ErCl_6_,^[^ ^8^ ^]^ Li_2_Sc_2/3_Cl_4_,^[^ ^9^ ^]^ doped halide conductors including Li_3−x_M_1−x_Zr_x_Cl_6_ (M = Y,^[^ ^10^ ^]^ Yb,^[^ ^11^ ^]^ Fe,^[^ ^12^ ^]^ Ho,^[^ ^13^ ^]^ Lu,^[^ ^10^ ^]^ In,^[^ ^14^ ^]^ Sc^[^ ^11^ ^]^), Li_3−x_Yb_1−x_M_x_Cl_6_ (M = Hf^4+^, Zr^4+^),^[^ ^15^ ^]^ and Li_3−x_In_1−x_Hf_x_Cl_6_,^[^ ^16^ ^]^ exhibit high oxidative stability to >4.2 V (versus Li/Li^+^) with ionic conductivities at around or above 1 mS.cm^−1^. However, electrochemical decomposition can still occur when they are paired with high‐voltage cathodes such as LiNi_0.85_Co_0.1_Mn_0.05_O_2_ (NCM85), which require charging to 4.3 V (versus Li⁺/Li) or higher to extract nearly full capacity. The degradation leads to the formation of a passivating, ion‐blocking interphase, which deteriorates battery performance.

Theoretical and experimental studies have shown that lithium metal fluorides offer high oxidative stability (>5 V), but their extremely low ionic conductivity limits their practical use.^[^ ^17^ ^]^ To address this problem, substituting Cl with F in chloride‐based SEs has emerged as a promising strategy. This dual‐halogen approach can potentially improve the oxidative stability of chloride SEs while maintaining suitable ionic conductivity. For instance, materials such as Li_3_InCl_4.8_F_1.2_,^[^ ^18^, ^19^ ^]^ Li_2_ZrCl_6−x_F_x_,^[^ ^20^, ^21^, ^22^ ^]^ Li_3−x_Y_1−x_Zr_x_Cl_6−z_F_z_ (0 ≤ *x* ≤ 0.75; 0 ≤ z ≤ 0.3),^[^ ^23^ ^]^ and Li_3_YBr_5.7_F_0.3_^[^ ^24^ ^]^ demonstrated significantly improved cell performance compared to their non‐fluorinated counterparts. Although these studies employed electrolytes with typically <20% fluorine content, the enhanced battery performance is often attributed to an expansion in the intrinsic electrochemical window (IEW).^[^ ^15^, ^16^, ^17^, ^20^, ^21^ ^]^ This raises an open question: is the improved performance indeed owing to an expanded IEW, or does improved interfacial compatibility between the SE and the cathode active material play a more dominant role?

In this work, we present a new family of SEs, Li_2_HfCl_6−x_F_x_, with good ionic conductivity, and investigate the impact of systematic fluoride substitution on their ionic and electronic conductivity, intrinsic electrochemical operating window, and overall battery performance to address this query. We find that although F substitution gradually expands the electrochemical window and beneficially decreases the electronic conductivity of the dual‐halogen electrolytes, it strongly decreases their ionic conductivity due to stronger coulombic interactions between F^−^ and Li^+^ ions. Therefore, heavily fluorinated chloride SEs suffer a significant drop in ionic conductivity and are not suitable as SEs. However, light fluorination offers an ideal compromise. All solid‐state full cells utilizing Li_2_HfCl_5.5_F_0.5_ as the catholyte cycled (2.8–4.3 V) at 0.2 C exhibit negligible capacity fade after 200 cycles at room temperature, compared to only 50% capacity retention for the Li_2_HfCl_6_ cell at a similar loading level. This marked enhancement in electrochemical behavior is primarily attributed to the formation of a kinetically stable LiF‐rich cathode electrolyte interphase (CEI), which suppresses side reactions between the NCM cathode and the SE, rather than to an expansion of the IEW. These insights are applicable to other dual‐halogen solid ionic conductors, shedding light on the interplay between IEWs, electronic and ionic conductivity, and their combined effect on battery performance in dual‐halogen systems.

## Results and Discussion

### Structural Evolution of Li_2_HfCl_6−x_F_x_

**Figure 1:**
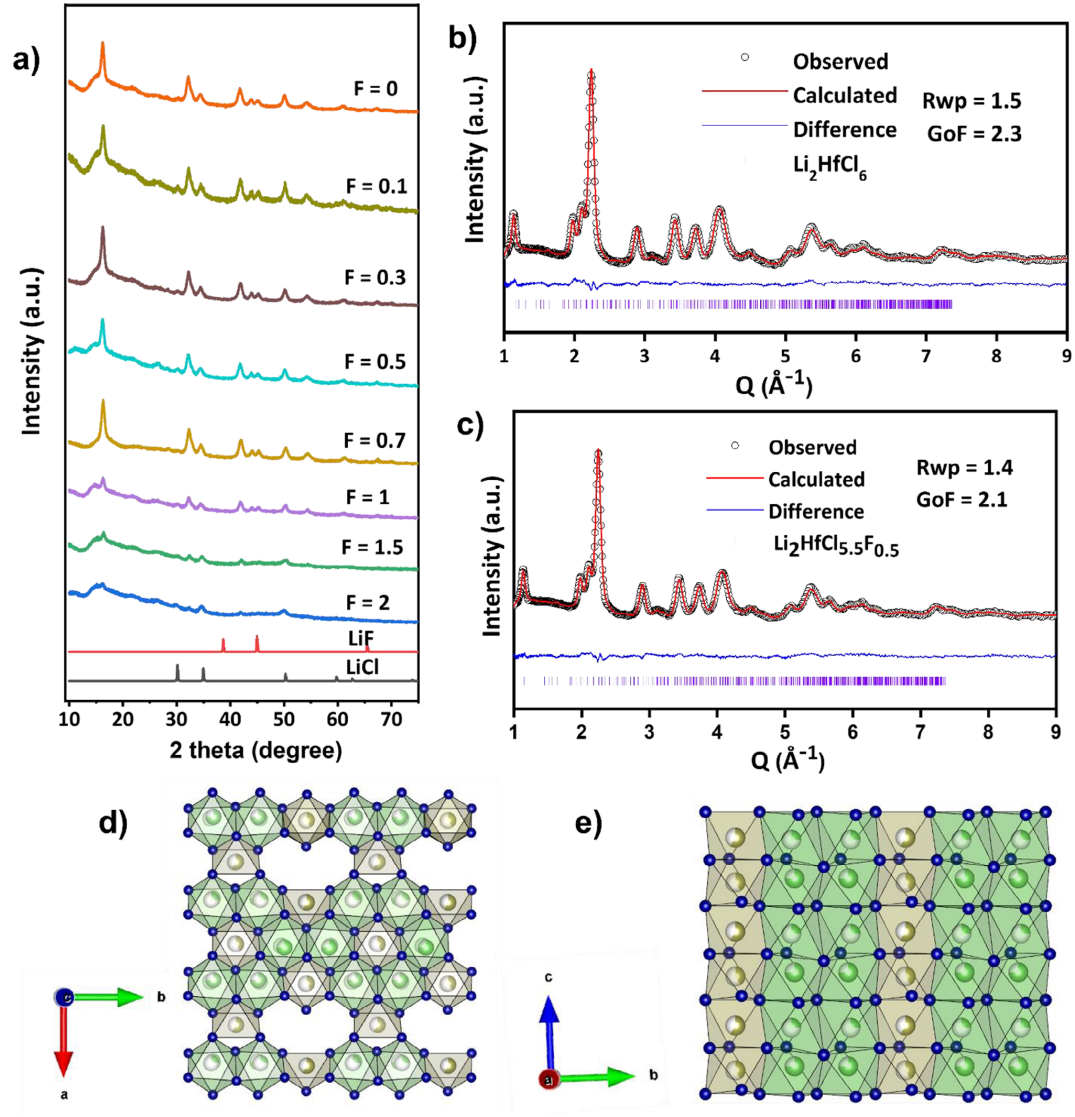
Structural characterization of Li_2_HfCl_6−x_F_x_. a) XRD patterns of Li_2_HfCl_6−x_F_x_ ((0 ≤ *x* ≤ 2). ToF powder neutron diffraction patterns and the corresponding Rietveld refinement of b) Li_2_HfCl_6_ and c) Li_2_HfCl_5.5_F_0.5_. Experimental data are represented by black circles, while the red line corresponds to the calculated pattern. The blue line indicates the difference profile, and the purple vertical ticks mark the calculated Bragg reflections. R_wp_ and GoF denote the weighted profile R‐factor and goodness of fit, respectively. The refined structure of Li_2_HfCl_6_ in the d) *ab* plane and e) *bc* plane.

X‐ray diffraction (Figure 1a) shows that synthesis of Li_2_HfCl_6−x_F_x_ (0 ≤ *x* ≤ 2) provides a phase‐pure orthorhombic phase that exists as a solid solution. Substitution of Cl with F for *x* < 1 shows no discernible changes in the diffraction pattern, and reflections of LiF or LiCl are not observed, suggesting that substitution was successful. At *x* > 1, the Bragg peaks become broader, and intensities merge into the background, indicating a lower crystallinity with higher F substitution.

The parent Li_2_HfCl_6_ and the fluorinated Li_2_HfCl_5.5_F_0.5_ structures were determined from Rietveld refinement against time‐of‐flight (ToF) neutron powder diffraction (Figure 1b,c) and X‐ray powder diffraction data (Figure S1). The relevant crystallographic details are summarized in Tables S1 and S2. Notably, the materials adopt a layered structure with a hexagonal close‐packed anion framework in the *Pnma* space group. Unlike other lithium metal halides with the *Pnma* structure, we identified two 4*c* Hf sites along the *c*‐axis, distinguishing it from other lithium metal halides with the orthorhombic‐I and orthorhombic‐II structures such as Li_3−_ *_x_* Zr*_x_* M_1−_ *_x_* Cl_6_ (M = Ho, Lu)^[^ ^10^ ^]^ and Li_3−_ *_x_* M_1−_ *_x_* Zr*_x_* Cl_6_ (M = Y, Er),^[^ ^7^ ^]^ synthesized by a simple heat‐treatment procedure. The two adjacent Hf sites both exhibit partial occupancies but only one site can be occupied within a single unit cell. A comparison of the R_wp_ and GoF values with varying Hf1 and Hf2 occupancies shows that introducing partial vacancies over both Hf sites leads to a distinctly improved fit for the disordered model (with 0.6 Hf1 occupancy) compared to either ordered motif (Figure S2). This resembles the cation ordering observed in Li_3_YCl_6_, recently reported by Kang and coworkers.^[^ ^25^ ^]^ The Hf/vacancies form an alternate zig‐zag ordering pattern in the *ab* plane, which is distinctly different from the previously reported Li_2_HfCl_6_ with the same stoichiometry. For instance, Tuo et al.^[^ ^26^ ^]^ assigned Li_2_HfCl_6_ to the *P*3̅*m*1 space group, whereas Li et al.^[^ ^27^ ^]^ attributed it to either *P3̅1c* or *P2/m*. However, we cannot index the X‐ray and neutron data for Li_2_HfCl_6_ in our study using any of these space groups. Li_2_HfCl_6_ features two distinct lithium sites, both occupying 8d positions but with differing occupancies of 0.27 and 0.73. The two Li sites both form LiCl_6_ octahedra surrounding Hf atoms, creating a honeycomb structure typical of other layered lithium metal chlorides. Two Li diffusion pathways exist in the structure, creating a three‐dimensional diffusion network. The first pathway lies in the *ab* plane, where Li^+^‐ions can hop between two sites through interconnected tetrahedra (Figure 1d). The second involves Li hops along the *c*‐axis between face‐shared Li octahedra (Figure 1e). Fluorinated Li_2_HfCl_5.5_F_0.5_ has a similar structure but with F distributed uniformly on the Cl sites. It is noteworthy that Zr⁴⁺ (0.72 Å) in octahedral coordination has an ionic radius nearly identical to that of Hf^4+^ (0.71 Å). However, ball‐milled Li_2_ZrCl_6_ is predominantly assigned to the trigonal (*P*3̅ *m*1) phase in the literature, raising the question of why this structural preference arises despite the similar ionic radii. X‐ray absorption spectroscopy and extended X‐ray absorption fine structure fitting results corroborate the neutron diffraction data, confirming the successful substitution of Cl by F. The local coordination environment around Hf remains unchanged upon fluorination, indicating that the favorable diffusion pathway is preserved (see Figure S3, Table S3, and Associated Discussion).

### Impact of Fluorination on Electrochemical Properties

**Figure 2:**
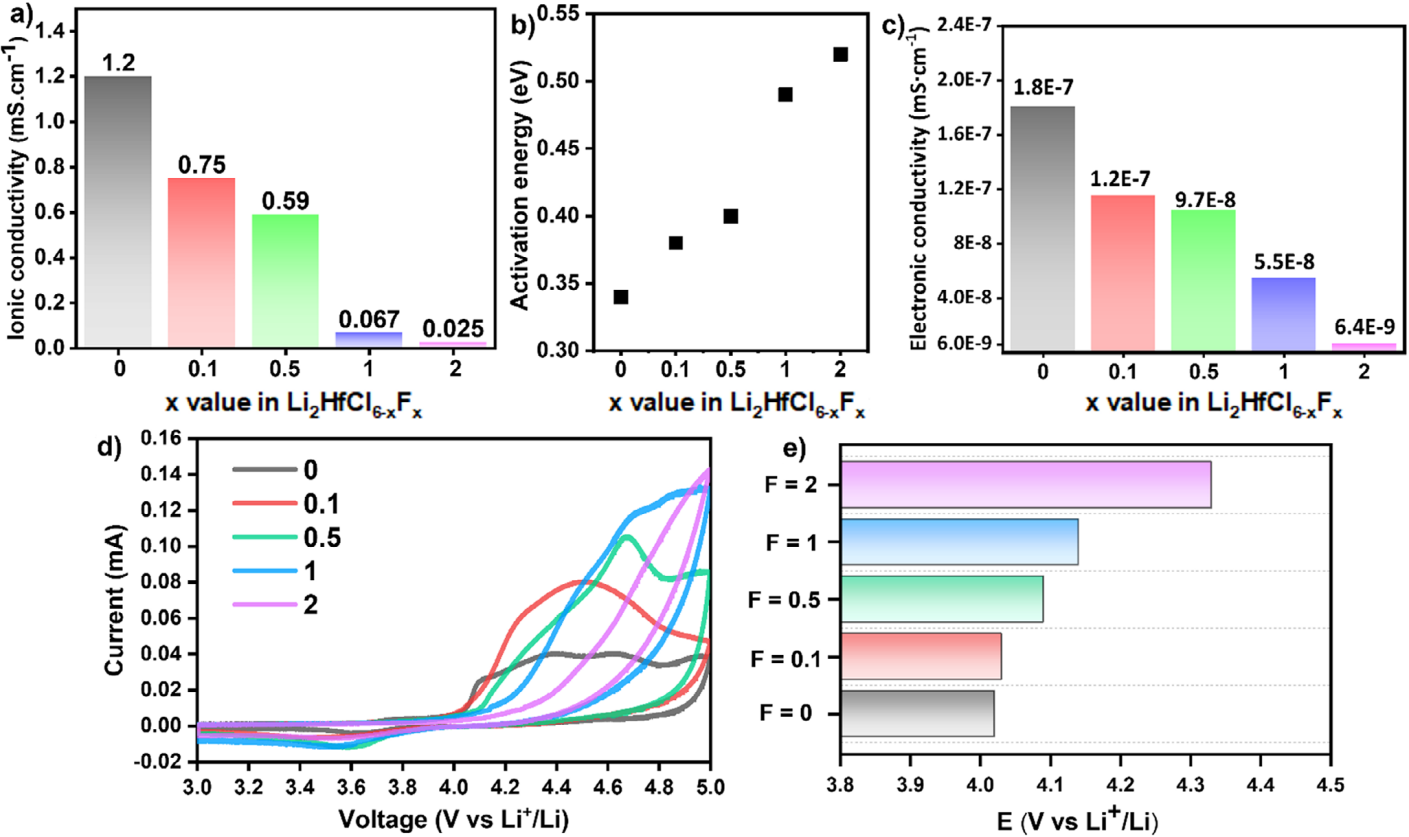
Electrochemical measurements of the Li_2_HfCl_6−x_F_x_ (0 ≤ *x* ≤ 2) materials. Summary of a) ionic conductivities, b) activation energies, and c) electronic conductivities. d) CV plots of the materials in carbon‐SE||In cells scanned from OCV to 5.0 V followed by a reverse scan to 3 V and return to OCV at 0.1 mV s^−1^. e) Summary of the onset electrochemical decomposition voltages as a function of fluorine substitution.

Temperature‐dependent electrochemical impedance spectroscopy (EIS) was utilized to determine the ionic conductivities, and activation energies for Li^+^ diffusion in Li_2_HfCl_6−x_F_x_ (0.14 < *x* ≤ 2) SEs. The results are summarized in Figure 2a,b, with the corresponding EIS spectra provided in Figure S4. Notably, our mechanochemically synthesized Li_2_HfCl_6_ demonstrated an ionic conductivity of 1.2 mS cm^−1^ at 30 °C, which is significantly higher than previously reported for this material (∼0.5 mS cm^−1^) with the same stoichiometry.^[^ ^23^ ^]^ This increase may be attributed to an apparent difference in structure and cation vacancies in our material (see discussion in Figure 1). Kang's work^[^ ^22^ ^]^ demonstrated computationally that an optimized partial occupancy of metal sites facilitates favorable lithium diffusion pathways and increases interlayer distances, which may also be the case for our synthesized Li_2_HfCl_6_. Upon substitution of Cl with F, Li_2_HfCl_6−x_F_x_ shows a consistent decrease in ionic conductivity (Figure 2a). Notably, when the F content reaches 1   , the ionic conductivity drops sharply, in line with the observed rise in activation energy (Figure 2b).

Electronic conductivities of Li_2_HfCl_6−x_F_x_ were measured through direct current polarization (Figure S5) and are summarized in Figure 2c. The electronic conductivity of Li_2_HfCl_6−x_F_x_ is 1.8 × 10^−7^ mS cm^−1^ at *x* = 0, and this drops by nearly 30‐fold to 6.4 × 10^−9^ mS cm^−1^ with increasing F substitution (up to *x* = 2). This reduction is attributed to the higher ionic character of the Hf─F bond compared to Hf─Cl. The smaller atomic radius and more localized electron density around the F atoms reduce orbital overlap between neighboring atoms, which hinders electron mobility.^[^ ^28^, ^29^ ^]^ The importance of low electronic conductivity in suppressing SE redox reactivity has been discussed in the literature,^[^ ^10^, ^30^ ^]^ and could play a minor role in explaining our improved battery performance (see later sections). The impact of fluorination on the IEW of Li_2_HfCl_6−x_F_x_ was evaluated using cyclic voltammetry (CV) performed on SE/carbon composites as the working electrode (Figure 2d). Incorporating sufficient carbon into the SE to enhance the Faradaic response is necessary, as shown by Zeier and coworkers.^[^ ^31^ ^]^ The onset voltages were evaluated by identifying the intercept point between the extrapolated anodic scan and the background, following our previous work.^[^ ^1^ ^]^ F doping raises the electrochemical oxidation stability of Li_2_HfCl_6−x_F_x_, as there is a consistent upshift of the onset decomposition voltage as the fluorine fraction increases. The variation in the CV profiles indicates differences in the oxidation process, potentially influenced by the higher kinetic barrier resulting from reduced electronic conductivity and improved IEW of the fluorinated SEs. However, the underlying mechanisms are complex and beyond the scope of this work. The onset electrochemical decomposition voltage values extracted from the CV are summarized in Figure 2e. We find that minor substitution of F with Cl (*x* < 0.5) results in a small increase in the anodic stability (<0.1 V), whereas a distinct enhancement (>0.3 V) is observed in the heavily fluorinated composition, Li_2_HfCl_4_F_2_. While the latter is undoubtedly due—at least in part—to a lower σ_e_, the difference in σ_e_ between *x* = 0 (1.8 × 10^−7^ mS cm^−1^) and *x* = 0.5 (9.7 × 10^−8^ mS cm^−1^) is only ∼1.8‐fold, which is unlikely to be a major factor. Figure S6 shows that after the initial cycle, the anodic peak nearly disappears, indicating the formation of a passivation layer at the interface between the carbon nanofiber and the SE.

### Electrochemical Performance

To examine the impact of fluorination, ASSBs were assembled using both Li_2_HfCl_6_ and fluorinated Li_2_HfCl_5.5_F_0.5_ as the SEs, Li–In alloy as the anode, and NCM85 as the cathode material. A previous study by Zeier and coworkers demonstrated chemical reactivity between the halide SE (Li_3_InCl_6_) and the Li_6_PS_5_Cl SE, especially when these SEs are in direct contact with the NCM cathode.^[^ ^32^ ^]^ Therefore, we employ a cell configuration (see details in Supporting Information), in which the composite cathode is placed on top of the halide SE, which serves as a separator from the underlying Li_6_PS_5_Cl layer positioned above the Li–In anode. This cell configuration mitigates the interfacial side reactions that could otherwise occur between the sulfide separator and the halide‐based cathode composite.^[^ ^32^ ^]^ Li_2_HfCl_5.5_F_0.5_ was selected for comparison with Li_2_HfCl_6_, as it preserves sufficient ionic conductivity while incorporating enough fluorine to assess the impact of fluorine substitution.

**Figure 3:**
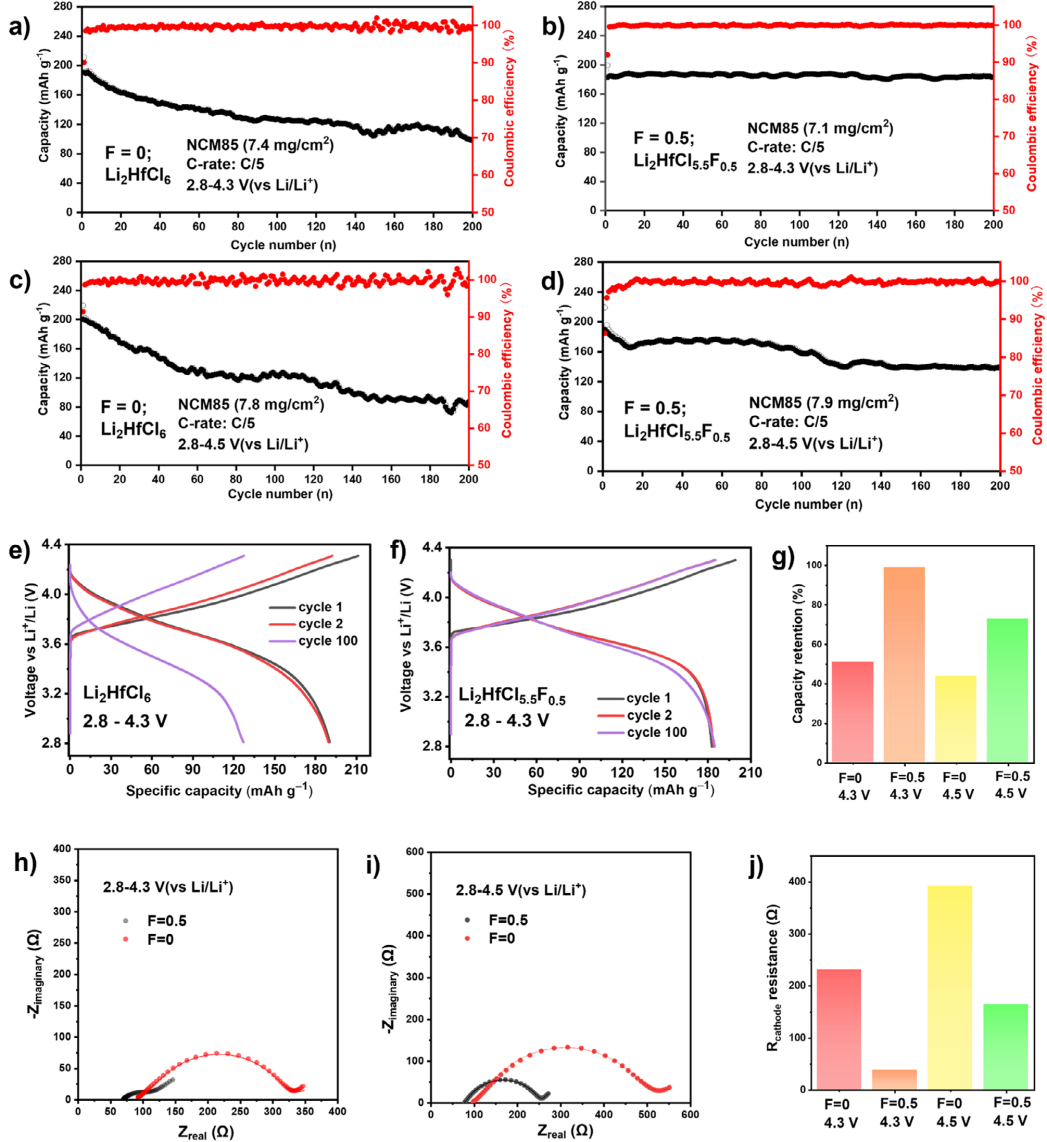
Electrochemical performance and impedance analysis of In/InLi||NCM85 ASSBs using Li_2_HfCl_6_ and Li_2_HfCl_5.5_F_0.5_ SEs under different cutoff voltages. Capacity retention of ASSBs cycled at C/5 C within the voltage windows of a), b) 2.8–4.3 V, and c), d) 2.8–4.5 V versus Li^+^/Li. Charge–discharge profiles of e) Li_2_HfCl_6_ and f) Li_2_HfCl_5.5_F_0.5_ cycled between 2.8–4.3 V versus Li^+^/Li, shown for the 1^st^, 2^nd^, and 100^th^ cycles. g) Summary of capacity retention after 200 cycles corresponding to (a–d). Nyquist plots for cells with upper cutoff voltages of h) 4.3 V and i) 4.5 V. j) Summary of resistance values extracted from (h, i).

Long‐term cycling at a C/5 rate was performed for cells utilizing Li_2_HfCl_6_ and Li_2_HfCl_5.5_F_0.5_, operating in a window between 2.8 and 4.3 V versus Li^+^/Li (Figure 3a,b). The corresponding voltage profiles are shown in Figure 3e,f. The Li_2_HfCl_6_‐catholyte cell displayed an initial discharge capacity of 190 mAh g^−1^, while the Li_2_HfCl_5.5_F_0.5_‐catholyte cell achieved a comparable capacity of 183 mAh g^−1^ at a cutoff voltage of 4.3 V. When the cutoff voltage was increased to 4.5 V (Figure 3c,d), the initial discharge capacity increased to 201 and 189 mAh g^−1^ in the case of Li_2_HfCl_6_ and Li_2_HfCl_5.5_F_0.5_ catholytes, respectively. The higher capacity of the Li_2_HfCl_6_ cells may be attributed to their higher ionic conductivity, which contributes to better charge transport within the cathode. Meanwhile, the capacity retention data clearly demonstrates the benefits of fluorination (Figure 3g). Cells with Li_2_HfCl_6_ retained only 51% capacity at a cutoff voltage of 4.3 V, while Li_2_HfCl_5.5_F_0.5_ exhibited negligible capacity fading. Raising the upper cutoff voltage to 4.5 V significantly decreases the cell stability for both electrolytes, but cells with Li_2_HfCl_5.5_F_0.5_ SE still demonstrate superior performance, retaining 73% capacity after 200 cycles compared to 44% for cells using the Li_2_HfCl_6_ catholyte.

Electrochemical impedance spectroscopy (EIS) was performed on cycled cells to evaluate the growth in cathode resistance resulting from the decomposition reactions between the SEs and NCM85. The Nyquist plots acquired after 200 cycles (Figure 3h,i) exhibit three distinct features: a high‐frequency onset (>10⁵ Hz), a semicircle between 10⁵ and 0.5 Hz, and a tail below 0.5 Hz. The value of the R_cathode_ for both coated and uncoated cathodes was determined using a modified transmission line model (Figure S7), as described in our previous work.^[^ ^1^ ^]^ For cells with a cutoff voltage of 4.3 V, R_cathode_ of the cell using Li_2_HfCl_6_ as SE (231 Ω) was approximately sixfold higher than that of the cell with Li_2_HfCl_5.5_F_0.5_ (38 Ω). The small R_cathode_ in the latter case aligns well with the superior battery performance observed in Figure 3b. Although raising the upper cutoff voltage to 4.5 V increases the R_cathode_ for both compositions, the Li_2_HfCl_5.5_F_0.5_‐based CAM exhibits a value approximately 2.5 times lower than its non‐fluorinated counterpart: 164 versus 392 Ω (Figure 3j). The increased degradation at the 4.5 V cutoff voltage could arise from increased reactivity between Hf^4+^ and the products of the oxygen evolution reaction in NCM85 at higher states of charge. At this potential, the H2‐H3 phase transition in NCM85 also induces significant contraction along the *c*‐axis, leading to structural collapse and causing intergranular cracking between particles, both of which accelerate capacity fade during cycling.^[^ ^33^ ^]^ In summary, cells using Li_2_HfCl_5.5_F_0.5_ as the SE demonstrated greatly superior battery performance compared to those using Li_2_HfCl_6_ as the SE The cycling stability of cells charged to 4.3 V using Li_2_HfCl_5.5_F_0.5_ show that it rivals some of the best reported SE materials.

### Origin of Enhanced Electrochemical Stability

As shown in Figure 2, heavily fluorinated chloride SEs exhibit a pronounced decrease in ionic conductivity, rendering them unsuitable for use as SEs. Thus, improved battery performance in dual‐halide systems typically involves only lightly fluorinated electrolytes (<10%), which retain sufficient ionic conductivity for practical applications. Nonetheless, consistent improvement in battery performance has been observed in these studies.^[^ ^15^, ^16^, ^19^ ^]^ As demonstrated in our work, such minor fluorine substitution should not significantly alter the inherent thermodynamic anodic stability, suggesting that other factors—such as interfacial compatibility—play a more critical role in enabling improved performance.

X‐ray photoelectron spectroscopy (XPS) was performed on the pristine SEs, and the cycled composite cathode was disassembled from cells using Li_2_HfCl_6_ and Li_2_HfCl_5.5_F_0.5_ as catholytes (cycled at cutoff voltages within 2.8 to 4.3 V at a C/5 rate after 200 cycles). In the Hf 4f XPS spectra of the pristine (uncycled) SEs (Figure S8)—in addition to the dominant signal from the Hf halide—a tiny component is assigned to HfO_x_ (Hf 4f_7/2_ at ∼17.6 eV) arising from slight moisture contamination in the chamber. After cycling, a very significant increase in that component is observed in the Hf 4f XPS for the Li_2_HfCl_6_ composite, indicating the formation of oxidized species arising from the interfacial reaction between CAM and the SE due to oxygen release from the NCM at elevated potential (Figure S9). In contrast, Li_2_HfCl_5.5_F_0.5_ shows virtually no increase in HfO_x_, suggesting that a passivated interface barrier exists that inhibits oxidation of the halide (see detailed discussion in Supporting Information).

Time‐of‐flight secondary ion mass spectrometry (ToF‐SIMS) was thus performed, leveraging its significantly higher sensitivity—several orders of magnitude above that of XPS—and its capability to detect and semi‐quantify molecular fragments.^[^ ^34^ ^]^ To account for surface inhomogeneity, five spectra were collected from various regions of each sample and averaged. The matrix effect can cause variations in the intensity of certain fragments (e.g., MCl_n_^−^), depending on the SE. To evaluate compositional changes at the interface, the normalized signal intensity of NCM85‐SE composite cathodes was compared before and after cycling.

**Figure 4:**
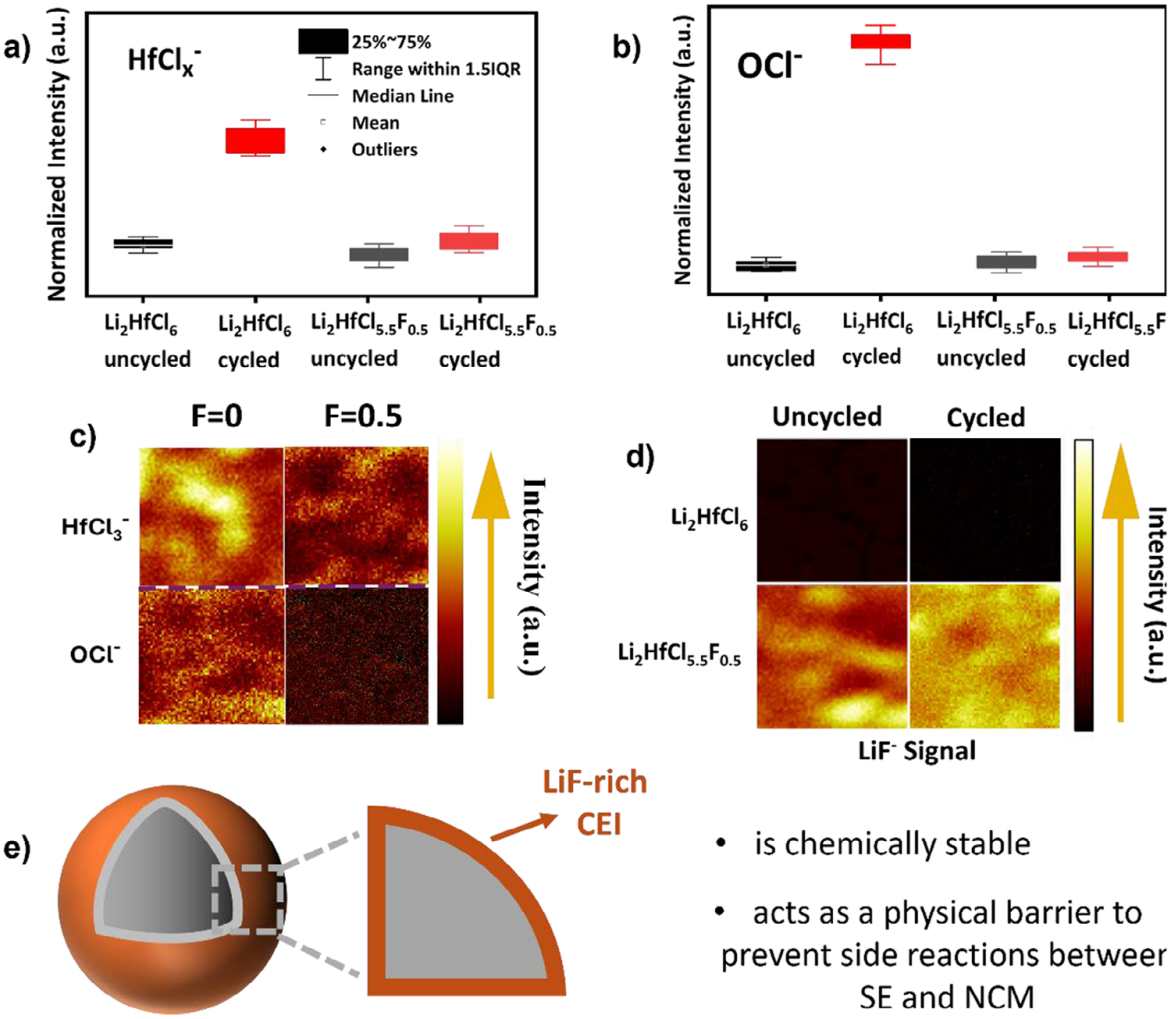
Degradation mechanism of the NCM and SE interface revealed by ToF‐SIMS measurements. Analyzed ToF‐SIMS results of normalized intensity of a) the HfCl_x_^−^ and b) the OCl^−^ fragments of uncycled cathode and cycled cathode composites within the cutoff potentials of 2.8 and 4.3 V, using Li_2_HfCl_6_ or Li_2_HfCl_5__.__5_F_0.__5_ as catholytes. c) Heat maps of HfCl_3_^−^ and OCl^−^ signals of the cycled composite. d) Heat maps of the LiF^−^ signals of uncycled and cycled NCM‐composites. e) Schematic representation of the role of LiF‐rich CEI in enhancing the battery performance.

It has been previously reported that intrinsic electrochemical degradation of the SE leads to the formation of metal chloride fragments (e.g., HfCl_x_^−^) and OCl^−^ fragments via chemical reaction between the chloride SE and NCM lattice oxygen evolved on charge to high potential.^[^ ^1^, ^27^, ^35^ ^]^ Indeed, Figure 4a shows an increase in HfCl_x_^−^ for the composite cathode with Li_2_HfCl_6_ after cycling. However, consistent with the higher oxidative stability and lower electronic conductivity of Li_2_HfCl_5.5_F_0.5_, cycling SSB cells with this catholyte does not significantly increase the HfCl_x_^−^ signal. Figure 4b compares the OCl^−^ fragments of the composite cathode before and after cycling. While the cells with Li_2_HfCl_6_ exhibit almost an 8‐fold increase in OCl^−^ signals after cycling, the cell with Li_2_HfCl_5.5_F_0.5_ does not result in any significant accumulation of OCl^−^ species. This is supported by the 2D reconstruction map shown in Figure 4c. The spatial distribution of these decomposition products reveals regions of heightened reactivity, likely corresponding to areas with high current density or localized stress within the electrode. Density functional theory (DFT) calculations predicting the enthalpy change and products (Table S4) of the interfacial reactions between Li_2_HfCl_6−x_F_x_ (*x* = 0, 0.5) and Li_0.25_NiO_2_ (a model system for high‐Ni NCM85 at the delithiated state) also show no significant difference. This suggests that F substitution does not limit the formation of OCl^−^ or HfO_2_ species between NCM85 and the SE. However, significant LiF fragments were observed in the ToF‐SIMS signal, which is consistent with our DFT results. 2D heat maps of the LiF^−^ fragments obtained from ToF‐SIMS results clearly reveal a significant increase in the LiF^−^ signal for the cycled Li_2_HfCl_5.5_F_0.5_ composite cathode compared to its uncycled counterpart (Figure 4d). This indicates that LiF forms as a prominent product of interfacial reactions between NCM85 and the Li_2_HfCl_5.5_F_0.5_ SE. 3D depth profile reconstruction (Figure S10) confirms the presence of LiF layers at the interfaces within the Li_2_HfCl_5.5_F_0.5_ composite cathode material. We propose that this interphase accounts for the excellent performance observed in Figure 3. The LiF‐rich CEI serves as a chemically stable barrier, preventing decomposition reactions between NCM and the SE (Figure 4e).

This aligns with previous reports indicating that F is effective in suppressing degradation reactions between high‐voltage cathodes and the electrolyte. For instance, Shen et al., using an F‐substituted oxychloride Li_2.5_ZrCl_5_F_0.5_O_0.5_ in conjunction with NMC in a solid‐state cell, recently reported the formation of a fluorine‐rich CEI on NMC that was comprised of NiF_2_.^[^ ^36^ ^]^ However, no evidence of LiF was presented. Using soft F K‐edge X‐ray absorption spectroscopy, Park et al. showed features of LiF on cycled lithium cobalt oxide cathodes in an SSB using Li_3_InCl_4.8_F_1.2_ as the catholyte.^[^ ^15^ ^]^ By comparison, our work clearly identifies in‐situ‐generated LiF as a major interfacial product between high‐Ni NCM and the dual‐halogen SE. The beneficial role of LiF as an ex situ coating in mitigating degradation reactions on high‐Ni NCM cathodes has been demonstrated in multiple studies. For instance, Wei et al. applied a thin LiF film onto NCM811 via magnetron sputtering and demonstrated improved battery performance, which was attributed to the suppression of decomposition reactions.^[^ ^37^ ^]^ Similarly, Llanos et al. employed atomic layer deposition to apply a conformal LiF coating, which led to significantly enhanced cycling stability.^[^ ^38^ ^]^ Together, these studies underscore the stabilizing role of LiF at the cathode‐electrolyte interface, whether applied artificially or formed in situ, as in our case. Furthermore, our ToF‐SIMS analysis shows suppression of the formation of OCl^−^ fragments in the cycled composite cathode, which suggests LiF acts as a physical barrier to prevent chemical reactivity between the SE and high‐Ni NCM. Our results suggest that oxygen release is suppressed from NCM85 because O^2−^ diffusion through LiF is negligible, as expected. We note that Persson and coworkers have shown that cathode coatings that have a slow O^2−^ diffusion rate can suppress oxygen loss from NCM cathode materials.^[^ ^39^ ^]^

## Conclusions

In this proof‐of‐concept work, we synthesized a family of Li_2_HfCl_6−x_F_x_ dual‐halogen SEs based on a new Li_2_HfCl_6_ *Pnma* phase with high ionic conductivity. Although F substitution extends the electrochemical window and decreases the electronic conductivity of the SE, it also reduces ionic conductivity due to the increased coulombic interactions between F^−^ and Li⁺ ions. Therefore, in most cases, we expect that heavily fluorinated chloride SEs will suffer a significant drop in ionic conductivity and are not suitable as SEs. However, light fluorination of the parent Li_2_HfCl_6_ to form Li_2_HfCl_5.5_F_0.5_ only resulted in a decrease in ionic conductivity from 1.2  to 0.6 mS cm^−1^—along with a beneficial lowering of the partial electronic conductivity—rendering this material a suitable candidate as a catholyte. The strong decrease in ionic conductivity upon fluorination implies that only highly conductive SEs will be suitable for this approach. The trade‐off between electrochemical stability and ionic conductivity must be carefully considered in the context of the specific application requirements, such as loading and C‐rate. This study provides direct guidelines on key parameters—including electronic conductivity, ionic conductivity, and cell stability—using this novel electrolyte as a representative example.

ASSBs assembled using Li_2_HfCl_5.5_F_0.5_ as the catholyte demonstrated significantly enhanced electrochemical stability compared to cells with Li_2_HfCl_6_. The Li_2_HfCl_5.5_F_0.5_ cell demonstrated nearly zero capacity fading when cycled to a cutoff voltage of 4.3 V, while the cell using Li_2_HfCl_6_ retained only 51% of its initial capacity. Significantly better performance was also observed even at a higher cutoff potential of 4.5 V versus Li^+^/Li. The excellent behavior is mainly attributed to the formation of an in‐situ‐generated LiF‐rich CEI (along with a slight increase in the anodic stability), which inhibits side reactions between the cathode and SE. Future studies will explore whether LiF also forms at the anode side when Li metal is used in conjunction with fluorinated chloride SEs, as LiF has been shown to suppress interfacial reactivity between SEs and the Li metal anode.^[^ ^40^, ^41^ ^]^ Moreover, replacing the Li–In alloy with Li metal would not only enhance the energy density but also reduce the overall cost of SSBs.

The findings from this study should be broadly applicable to other dual‐halogen solid ionic conductors, providing valuable insights into the interplay between intrinsic electrochemical stability, electronic and ionic conductivity, and battery performance in these materials.

## Author Contributions

L.F.N. and L.Q. conceived the concept and designed the experimental work. J.L. collected the neutron diffraction data. L.Q., Y.W., and J.L. carried out the structural refinement. L.Q. collected the hard X‐ray XAS results at the CLS. N.C. and L.Q. performed the EXAFS fitting. C.D. conducted the DFT calculations. L.Q and Y.W. acquired all the electrochemical results. L.Q and I.K. fitted and analyzed the XPS data. L.Q. and L.F.N. wrote the manuscript with input from all the authors. L.F.N supervised the work.

## Conflict of Interests

The authors declare no conflict of interest.

## Supporting information



Supporting Information

## Data Availability

The data that support the findings of this study are available in the Supporting Information of this article.
