# Supplementary material for: Deciphering the Role of Fluorination in Dual‐Halogen Electrolytes for All‐Solid‐State Batteries: A Case Study of New Li2HfCl6−xFx Solid Electrolytes
Source: Angew Chem Int Ed Engl. 2025 Aug 20;64(41):e202509209. doi: 10.1002/anie.202509209 (PMC12501681; doi:10.1002/anie.202509209)
Supplement: Supplementary file 1 — Supporting Information [file ANIE-64-e202509209-s001.docx]

***Supporting Information***

**Deciphering the Role of Fluorination in Dual Halogen Electrolytes for All-Solid-State Batteries: A Case Study of New Li_2_HfCl_6-x_F_+x_ Solid Electrolytes**

Lanting Qian^a,b^, Yubo Wang^a^, Jue Liu^c^, Ivan Kochetkov^a^, Ning Chen^d^, Cameron Dean^a^, Linda F Nazar^a,b*^

1. Department of Chemistry, Waterloo Institute of Nanotechnology, University of Waterloo, Ontario, N2L 3G1, Canada.
2. Department of Chemical Engineering, University of Waterloo, Ontario, N2L 3G1, Canada.
3. Neutron Scattering Division, Oak Ridge National Laboratory, Oak Ridge, Tennessee 37822, United States.
4. Canadian Light Source, 44 Innovation Blvd, Saskatoon, SK, S7N 2V3, Canada.

**Methods**

*Materials Synthesis*

Stoichiometric amounts of commercial HfCl₄, LiCl, and LiF (Sigma-Aldrich, > 99.99%) were mixed in an argon-filled glove box (MBraun, H₂O, O₂ < 0.5 ppm). The mixture was then transferred into an 80 mL zirconia ball mill jar containing approximately 120 g of 5 mm diameter ZrO₂ balls. The jar was sealed within the glove box and subsequently transferred to a planetary ball mill (Fritsch PULVERISETTE 7 Premium). The materials were milled at 700 rpm for 30 hours, yielding Li_2_HfCl_6-x_F_x_.

*Cell Assembly*

LiNi_0.85_Mn_0.1_Co_0.05_O_2_ (NMC85, D_50_ ~ 4 µm) was sourced from BASF, while Li foil was obtained from Sigma-Aldrich and In foil from CMR-direct. All-solid-state batteries were assembled using Li_2_HfCl_6-x_F_x_ solid electrolytes, NCM85 cathodes, and a commercial Li_6_PS_5_Cl separator positioned between the solid electrolyte and the Li-In anode. The assembly procedure is detailed as follows: A PEEK cylinder was filled with approximately 140 mg of Li_6_PS_5_Cl powder and compressed at 250 MPa for one minute to form a pellet. Subsequently, 50 mg of Li_2+x_HfCl_6-x_F_x_ SE were placed on top of the Li_6_PS_5_Cl pellet and compressed at 250 MPa for 3 minutes. Composite cathode mixtures were created by grinding Li_2+x_HfCl_6-x_F_x_ electrolytes with NCM85 in an agate mortar at a weight ratio of 2:8 for about 40 minutes. The resulting cathode composites were spread on top of the halide SE pellet and compressed at 200 MPa for approximately 3 minutes. A thin indium foil (10 mm in diameter and 0.1 mm thick) was affixed to the opposite side of the Li_6_PS_5_Cl pellet. Approximately 1.5 mg of lithium, pre-flattened onto copper foil, was placed over the indium foil to form the LiIn alloy anode. Finally, a load cell was employed to measure the initial 200 MPa applied pressure before enclosing the entire assembly in a stainless-steel casing.

*Cyclic Voltammetry*

80 mg of Li_6_PS_5_Cl powder (MSE Supplies LLC, D50 ~ 1 μm) was placed into a PEEK cylinder and compressed at 250 MPa for 1 minute to form a pellet with a 10 mm diameter for CV measurements. Subsequently, 50 mg of solid electrolyte powder was evenly distributed over one side of the Li_6_PS_5_Cl pellet and pressed at 250 MPa for an additional minute. The SE-carbon nanofiber composite electrode was prepared by mixing SE and carbon nanofiber in a weight ratio of 9:1, followed by hand-grinding the mixture in an agate mortar for 25 minutes. The Li-In electrode is fabricated in a similar manner described above for cell assembly. Finally, a load cell was employed to measure the initial 200 MPa applied pressure before enclosing the entire assembly in a stainless-steel casing. The voltammetry measurement was performed using a VMP3 potentiostat (Bio-Logic) at a scan rate of 0.1 mV·s⁻¹.

*Powder X-ray Diffraction*

Powder X-ray diffraction (XRD) measurements were conducted at room temperature using a PANalytical Empyrean diffractometer equipped with Cu–Kα radiation and a Pixcel bidimensional detector. Samples were mounted on a zero-background silicon holder in the glove box and sealed with Kapton film to prevent moisture contact with the powder. For X-ray diffraction data used for Rietveld refinement, the powder was sealed in a quartz capillary tube inside the glove box and mounted on the diffractometer in the spinning capillary configuration to minimize orientation effects. Data collection was carried out overnight.

*Time of flight Neutron Powder Diffraction*

Neutron scattering data were collected at the Nanoscale-Ordered Materials Diffractometer (NOMAD) beamline at the Spallation Neutron Source at Oakridge National Lab. Quartz capillaries (3 mm diameter) were used as the sample holders. Four 24 min scans (2C proton charge each) were collected and summed together for each sample to improve the statistics. The background was corrected by subtracting the scattering signal from an empty 3 mm quartz capillary. The background-subtracted scattering data were then normalized by the scattering intensity from a 6 mm vanadium rod to correct for the detector efficiency and incident neutron beam profile. For structure refinements using neutron Bragg diffraction data, time-of-flight (TOF) data were converted to d-spacing data using the second order polynomial TOF = ZERO + DIFC*d + DIFA*d,4 where ZERO is a constant, DIFC is the diffraction constant. During the structure refinement, ZERO and DIFC were determined from refinement of a standard NIST Si-640e or NAC data set and held fixed, while DIFA, the moderator induced line profile, was modeled using a modified Ikeda-Carpenter-David function.^4, 5^ The Lorenz factor was corrected by multiplying (d_spacing)^4. The isotropic instrument broadening effect was modeled by convoluting it with a Pseudo-Voigt function. The instrument peak profiles were fixed during structure refinement, while the sample induced peak broadening (microstrain and size broadening) terms were refined.

*Structure Solution and Rietveld Refinement*

Rietveld refinements were carried out in TOPAS v6.8. For ab initio structure solution, powder XRD data were first indexed. A Pawley fit was then used to extract the corresponding structure factor modular. Charge flipping was then used to identify the potential Hf and Cl positions in the structure. Fourier difference maps from neutron diffraction data were used to identify the potential Li sites in the structure. The final Rietveld refinement was carried out against the powder neutron diffraction data. For the Li_2_HfCl_6_ and Li_2_HfCl_5.5_F_0.5_ materials, we found that the common orthorhombic space group *P*nma can well index all Bragg diffraction peaks, while the *P*3̅m1, *P*3̅1c or *P*2/m cannot fully index all the peaks. Therefore, a structure model from the orthorhombic Li_3_YCl_6_ was used as the starting point for the Rietveld refinement.

*X-ray Photoelectron Microscopy and Time-of-Flight Secondary Ion Mass Spectrometry*

X-ray photoelectron spectroscopy (XPS) was performed using a Thermo Fisher K-Alpha XPS system equipped with dual turbo molecular pumps and a monochromated, micro-focused, low-power Al K-Alpha X-ray source, along with a 128-channel detector for rapid data acquisition. Time-of-Flight Secondary Ion Mass Spectrometry (ToF-SIMS) spectra were obtained using an ION-TOF ToF-SIMS 5 instrument (Münster, Germany), operated in negative-ion mode with a 30 keV cluster primary ion gun over a 200 × 200 μm² area, rasterized at 256 × 256 pixels. The principal ion current was approximately 0.3 pA, and the stop condition was set to 5 × 10¹² ions per cm² to enable semiquantitative analysis. For depth profiling, a Ga⁺ ion sputtering gun was employed, allowing ToF-SIMS data to be collected layer by layer for 3D reconstruction. Data evaluation was conducted using SurfaceLab v.7.2 software (IONTOF). Secondary-ion signals were normalized to the total ion signal, with all intensity data collected from the corresponding normalized secondary-ion images to ensure accurate relative intensity measurements.

*Electrochemical Measurements*

Room temperature ionic conductivity was measured via electrochemical impedance spectroscopy (EIS) using a sinusoidal excitation voltage amplitude of 100 mV, within a frequency range of 1 MHz to 50 mHz. For activation energy measurements, impedance was recorded across the same frequency range with a sinusoidal excitation voltage amplitude of 100 mV, at temperatures ranging from 15°C to 45°C. The ionic conductivity (σ_i_) was calculated using the formula: σ_i_ = L / (Rb × A), where σ_i_ is the ionic conductivity (S·cm⁻¹), L is the pellet thickness (cm), and A is the contact area (cm²). The activation energy (E_a_) of the electrolytes was derived from the Arrhenius equation: σT = σ₀ exp (-E_a_/kBT), where σ is the ionic conductivity (S·cm⁻¹), T is the temperature (K), σ₀ is the pre-exponential factor, E_a_ is the activation energy in eV, and kB is the Boltzmann constant. A Bio-logic SP-200 potentiostat with an ultra low current option was used to accurately measure low currents via DC polarization, by applying a constant voltage of 0.5 V for 10 hours to achieve a steady state current. The electronic conductivity (σ_e_) was calculated using Ohm’s law: σe = I_e_ × L / (V × A), where σ_e_ is the electronic conductivity, I_e_ is the equilibrium current, L is the material thickness, V is the applied voltage, and A is the material area. The cells were galvanostatically cycled at room temperature using either a VMP3 (Bio-Logic) or MACCOR cycler, with the voltage range and cycling rate outlined in the manuscript. EIS measurements were taken at 25°C using a Bio-Logic SP-200 potentiostat, over a frequency range of 100 mHz to 1 MHz, and the data were fitted using RelaxIS software.

*X-ray Absorption Spectroscopy*

Hard X-ray XANES and EXAFS data at the Hf L-edge were collected from the Hard X-ray Micro Analysis (HXMA) beamline at the Canadian Light Source (CLS). The detector used was a 32 element Ge detector with a 63-pole superconducting wiggler. Background subtraction and normalization were performed using Athena, while EXAFS fitting and modeling were conducted with WinXAS and Artemis, utilizing FEFF and FDMNES codes. The initial model was acquired from the solved structure acquired from neutron and X-ray diffraction. Wavelet transform analysis of the k³-weighted EXAFS spectra in k-space was carried out based on the Morlet wavelet. Soft X-ray spectra including F K-edge and Hf M-edge were collected at the Spherical Grating Monochromator (SGM) beamline using a four-element silicon drift detector array. Both partial fluorescence yield and total electron yield data were simultaneously collected. The samples were transferred through a sealed chamber from the glovebox to ensure an air-free environment. Data processing was performed with Athena.

*Computational Methods*

Density functional theory (DFT) calculations were performed using the Vienna Ab Initio Simulation Package (VASP)^1,2^, which implements the projector augmented wave (PAW) method. In this approach, electron orbitals are represented by plane waves, and core electrons are approximated using pseudopotentials. The exchange-correlation functional was described using the Perdew–Burke–Ernzerhof (PBE) formulation of the generalized gradient approximation (GGA)^3,4^.

**Simulation of Li_2_HfCl_6-x_F_x_ Structure:** The parent Li_2_HfCl_6_ structure was obtained through Rietveld refinement of neutron and X-ray powder diffraction data, revealing partially occupied Li and Hf sites. Ordering of Li and Hf was conducted using a three-step process employing the pymatgen^5^ and matgl^6^ Python packages. Initially, pymatgen’s implementation of enumlib^7^ was utilized to generate all unique occupancy combinations. These configurations were ranked by Ewald energy, with the 500 lowest-energy structures selected for further evaluation. Subsequently, matgl’s Materials 3-body Graph Network (M3GNet)^8^, employing a pre-trained universal interatomic potential, was applied to perform ionic relaxations on these 500 ordered structures. The ten structures with the lowest M3GNet energies, along with five additional structures exhibiting diverse Hf orderings, were chosen for detailed density functional theory (DFT) calculations to ensure completeness. These selected structures underwent relaxation in VASP using a plane-wave cutoff energy of 520 eV and a Monkhorst-Pack k-point mesh with a minimum density of 699/N, where N represents the total number of atoms. Total energies were converged to within 10⁻⁵ eV, and atomic forces were converged to within 0.01 eV/Å. The structure exhibiting the lowest energy was adopted as the representative ordered Li₂HfCl₆ configuration. An iterative approach was employed to determine the structures of F-doped Li₂HfCl_6-x_F_x_. In each iteration, the energetics of a single F-for-Cl substitution were assessed using DFT. In the initial iteration, 24 possible Cl substitution sites were evaluated, and the lowest-energy Li₈Hf₄Cl₂₃F structure was selected as the representative x=0.25 configuration. The process continued iteratively, reducing the available Cl sites by one each step. The lowest-energy configurations for x = 0.5 (Li₈Hf₄Cl₂₂F₂), and subsequently for x = 0.75, 1.0, 1.25, 1.5, 1.75, and 2.0, were identified following this method. Final structure optimizations for each composition used identical DFT parameters are complemented by the DFT-D3 van der Waals dispersion correction from Grimme^9^.

**Interfacial Reactivity Calculation:** The thermodynamically favourable decomposition products between Li_2_HfCl_6-x_F_x_ (x = 0,0.5) and Li_0.25_NiO_2_ were calculated using pymatgen’s interfacial reactivity class^10^. This method uses all computed entries in the given chemical system in the Materials Project Database^11^ to predict the chemical species that minimize the formation energy of the interphase as a function of the fractional component of each bulk material. This leads to the identification of several reactions that occur within the phase diagram of these materials, and the reaction with the lowest energy was identified as the most favourable interfacial reaction. The Li_2_HfCl_6-x_F_x_ structures were identified using the method described above, and their energies were re-calculated using parameters that match the Materials Project for appropriate comparison.

**References**

[1] G. Kresse, J. Hafner, Ab initio molecular dynamics for liquid metals. *Phys. Rev. B.* 1993, 47, 558; ibid. 1994, 49, 14251.

[2] G. Kresse, J. Furthmüller, Efficient iterative schemes for ab initio total-energy calculations using a plane-wave basis set. *Comput. Mat. Sci.* 1996, 6, 15.

[3] ​M. Bursch, J.-M. Mewes, A. Hansen, S. Grimme. Best-Practice DFT Protocols for Basic Molecular Computational Chemistry. *Angew. Chem. Int. Ed.* 2022, 61, e202205735.

[4] T. H. Dunning Jr., “Gaussian basis sets for use in correlated molecular calculations. I. The atoms boron through neon and hydrogen,” *J. Chem. Phys*., 90 (1989) 1007-23. DOI: 10.1063/1.456153

[5] S.P. Ong, W.D. Richards, A. Jain, G. Hautier, M. Kocher, S. Cholia, D. Gunter, V. Chevrier, K.A. Persson, G. Ceder, Python Materials Genomics (pymatgen): A Robust, Open-Source Python Library for Materials Analysis. *Comput. Mat. Sci.* 2013, 68, 314–319.

[6] MATGL Team. Materials Graph Learning (MATGL): A Cutting-Edge Open-Source Framework for Graph-Based Materials Analysis. MATGL.ai, 2025. https://matgl.ai/ (accessed March 25, 2025).

[7] Enumlib: A Library for Enumerating Symmetry-Distinct Atomic Configurations; GitHub Repository. https://github.com/msg-byu/enumlib (accessed March 25, 2025).

[8] C. Chen, S.P. Ong. A universal graph deep learning interatomic potential for the periodic table. *Nat. Compu. Sci*. 2022. 2, 718-728.

[9] S. Grimme, J. Antony, S. Ehrlich, H. K. A consistent and accurate ab initio parametrization of density functional dispersion correction (DFT-D) for the 94 elements H-Pu. *J. Chem. Phys*. 2010. 132, 154104.

[10] W.D. Richards, L.J. Miara, Y. Wang, J.C. Kim, G. Ceder. Interface stability in solid-state batteries. *Chem. Mater.* 2015, 28, 266-273.

[11] A. Jain, S. P. Ong, G. Hautier, W. Chen, W. D. Richards, S. Dacek, S. Cholia, D. Gunter, D. Skinner, G. Ceder, K. A. Persson. Commentary: The Materials Project: A materials genome approach to accelerating materials innovation. *APL Mater* 2013, **1**, 011002.

[12] C. Sleigh, A.P. Pijpers, A. Jaspers, B. Coussens, R. J. Meier. On the determination of atomic charge via ESCA including application to organometallics. *J. Electron. Spectroscopy and Related Phenomena*, 1996, 77, 41-57.

**List of Supplemental Figures**

**
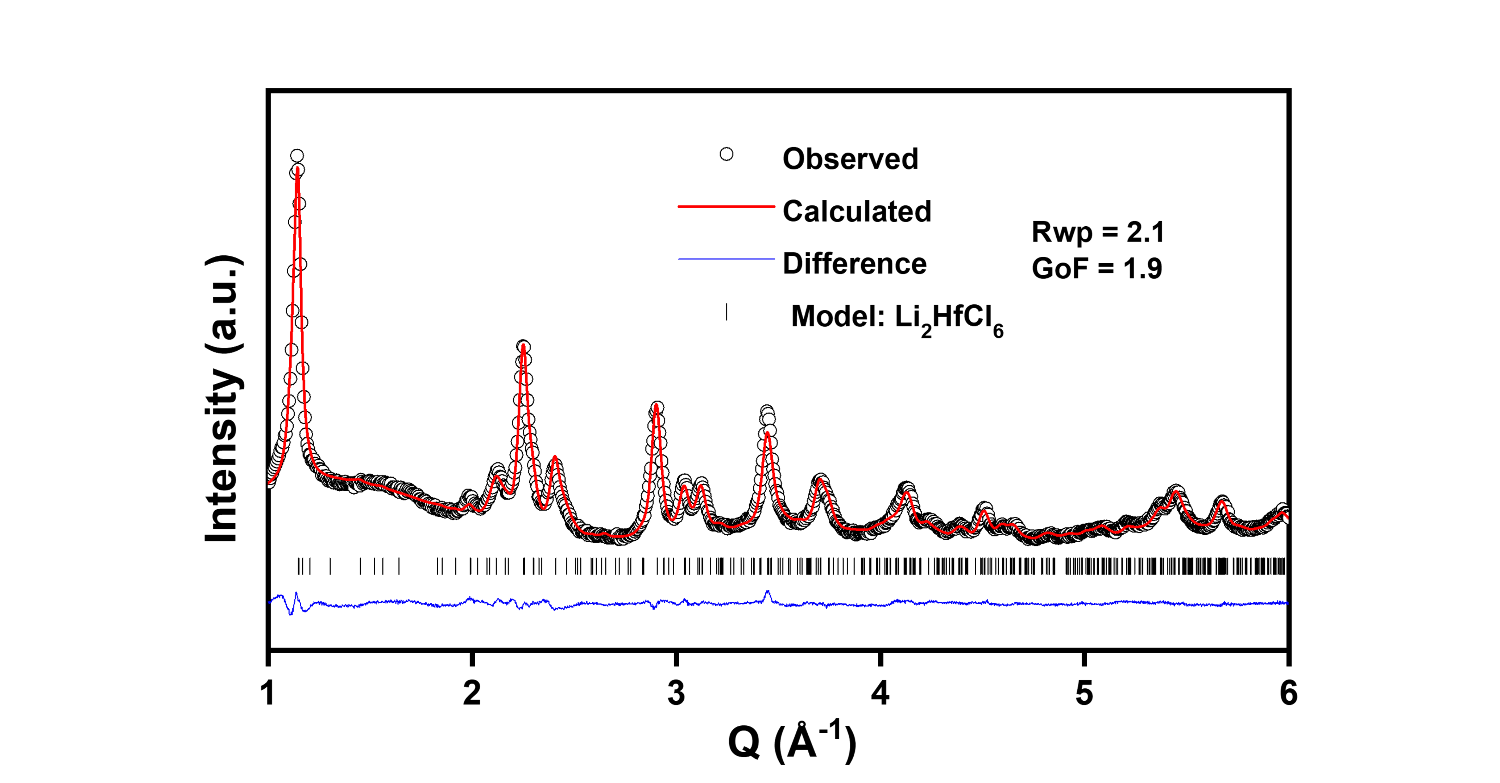
**

**Figure S1.** Rietveld refinement of the Li_2_HfCl_6_ X-ray powder diffraction pattern obtained from a lab X-ray source in a spinning capillary set up to minimize any orientation effects. Experimental data are shown in black circles; the red line denotes the calculated pattern; the blue line denotes the difference profile, and the calculated Bragg reflections are represented by the black vertical ticks. R_wp_ and GoF are the weighted profile R-factor and goodness of fit respectively.


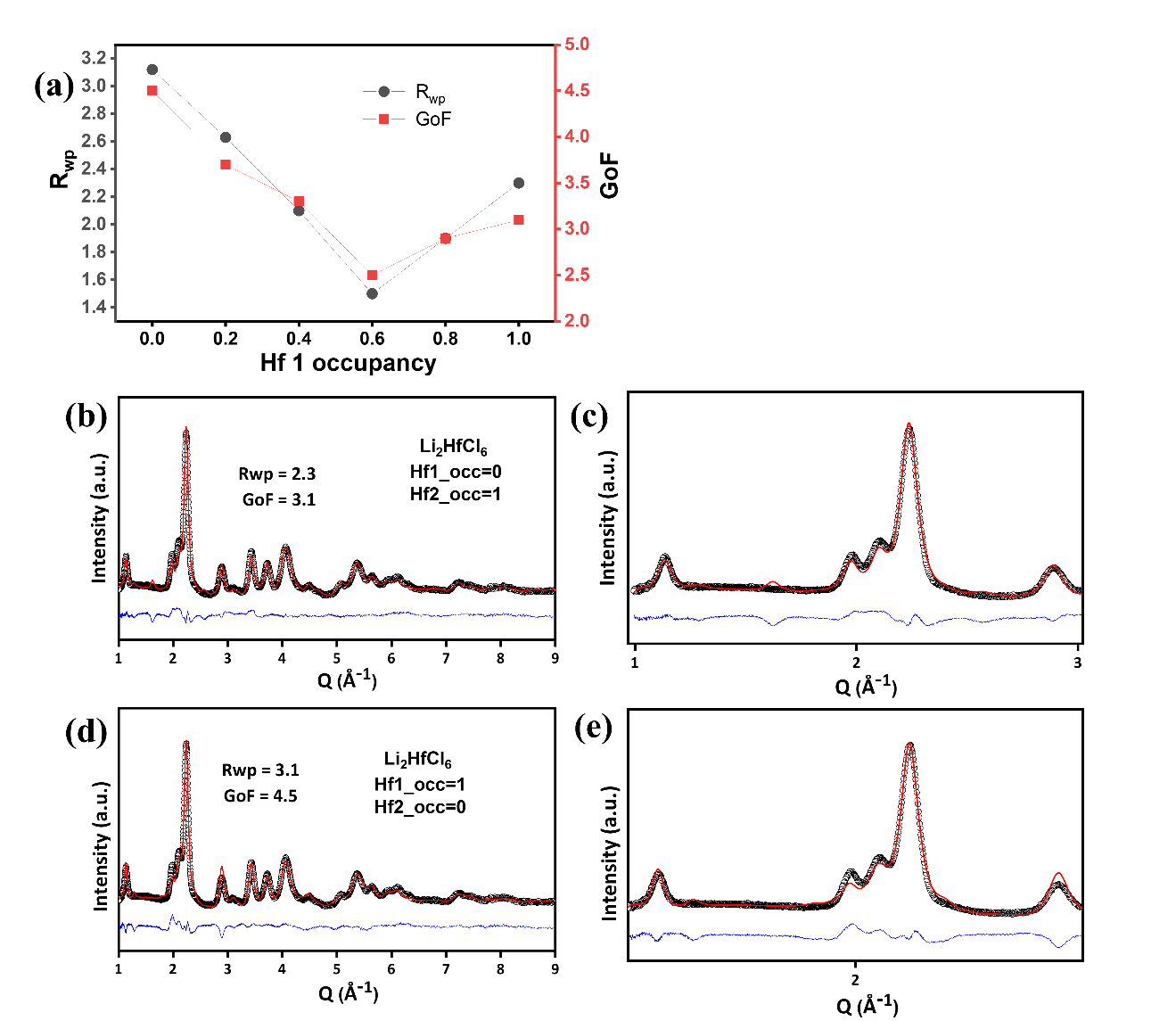


**Figure S2.** Comparison of Rietveld refinement data with varying Hf site occupancies. (a) Summary of R_wp_ and GoF with varying Hf1 and Hf2 occupancies. (b) Rietveld refinement assuming full occupancy at the Hf2 site. (c) Rietveld refinement assuming full occupancy at the Hf1 site.The disordered model—featuring partial occupancy on both Hf sites (**Fig S2a**)—yielded a lower *weighted* R-factor (R_wp_ = 1.5) and improved goodness-of-fit (GoF = 2.1) relative to the fully ordered structures (full occupancy on either Hf2 — (b,c) or on Hf1 —(d,e) — indicating a better fit to the experimental data. This indicates that Hf exhibits a preference for partial occupancy between distinct crystallographic sites, indicative of cation disorder in Li_2_HfCl_6_. Such disorder may influence ionic conductivity through subtle changes in local environments and Li⁺ migration pathways.

**
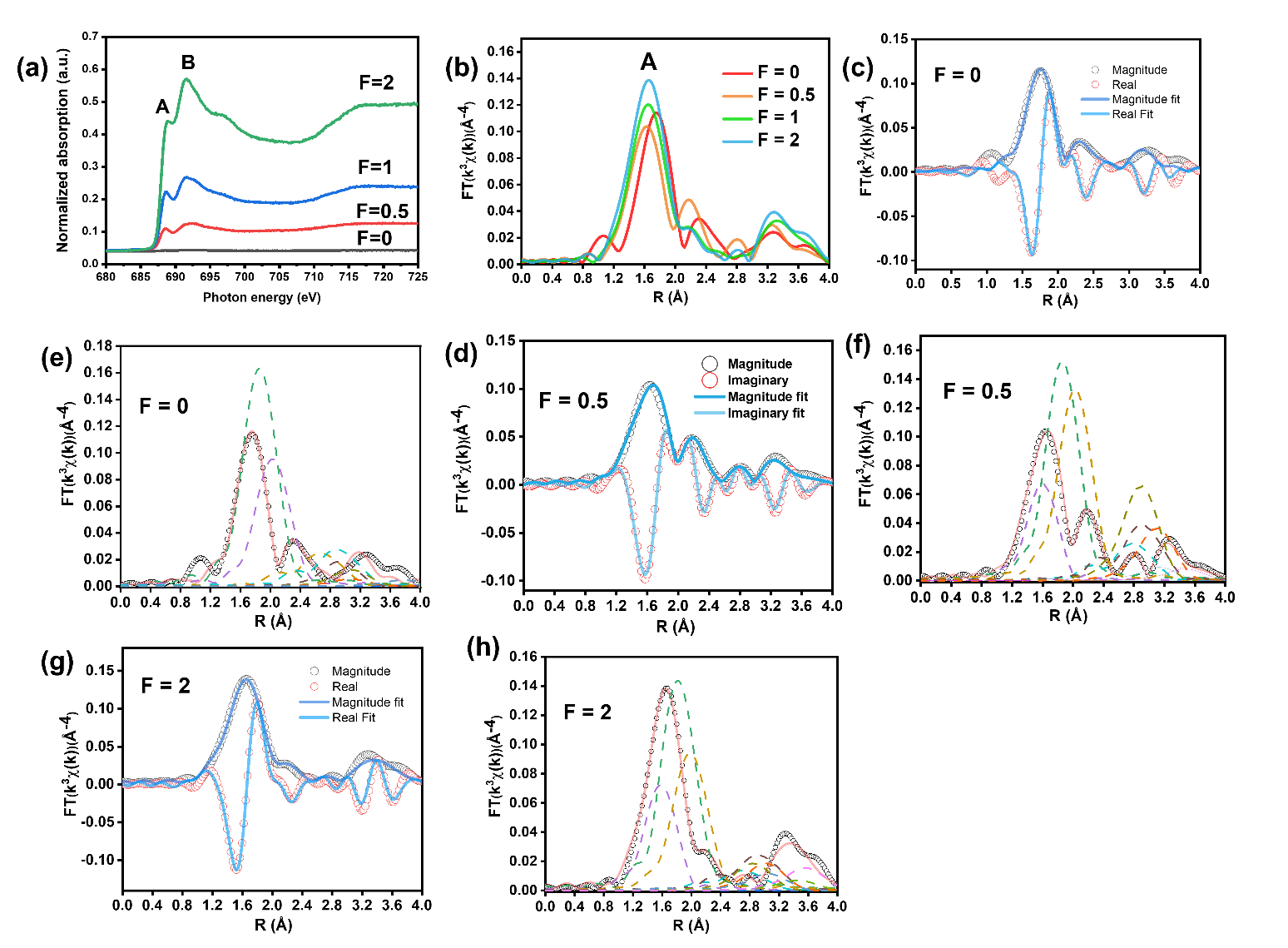
Figure S3.** XAS analysis of the local structure of the Li_2_HfCl_6-x_F_x_ compositions. (a) F K-edge of Li_2_HfCl_6-x_F_x_ materials. The F-Kedge spectra directly probe the ligand (F 2p states). The intense absorption band labelled as A (~688 eV) originates from the transition of F 1s electrons to the lowest unoccupied electronic states of a_1g_ and t_1u_ symmetry. Peak B originates from occupied electronic states corresponding to the hybridization of the Hf 5d orbitals with the F 2p orbitals. The observed increase in intensity is directly correlated with a higher degree of F substitution for Cl. (b) *k*^3^-weighted (phase uncorrected) of XAS spectra in R space. Fitted results of magnitude and imaginary part of the EXAFS data for (c) Li_2_HfCl_6_, (e) Li_2_HfCl_5.5_F_0.5_, and (g) Li_2_HfCl_4_F_2_. The Individual scattering path of (d) Li_2_HfCl_6_, (f) Li_2_HfCl_4_F_2_, (h) Li_2_HfCl_4_F_2_. The fitting results indicate successful substitution of Cl with F, accompanied by only negligible changes in the coordination environment around Hf.

***
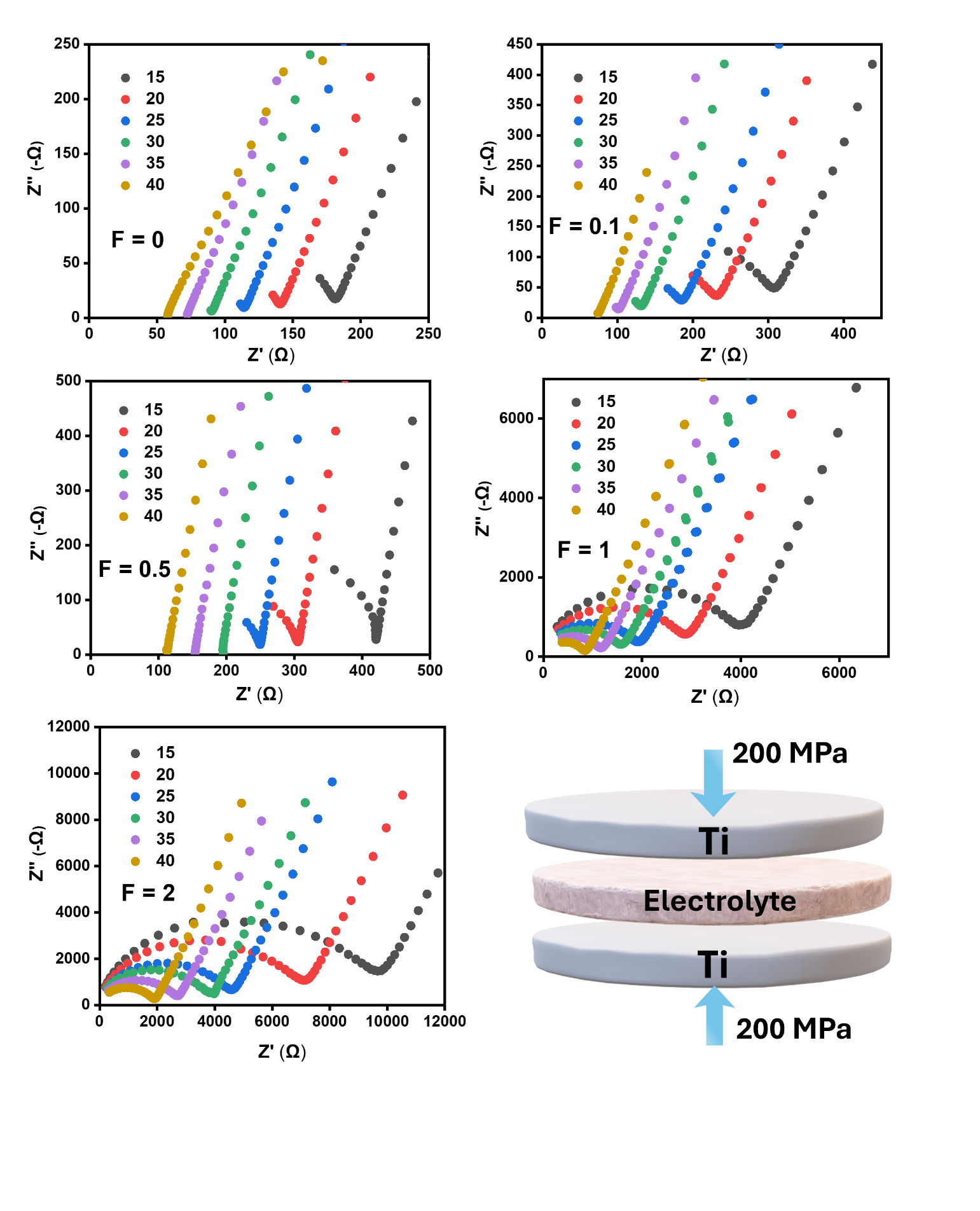
***

**Figure S4.** Nyquist plots at temperatures from 15 °C to 40 °C, which were used to extract the ionic conductivity values for activation energy calculations of Li_2_HfCl_6-x_F_x_ materials.

***
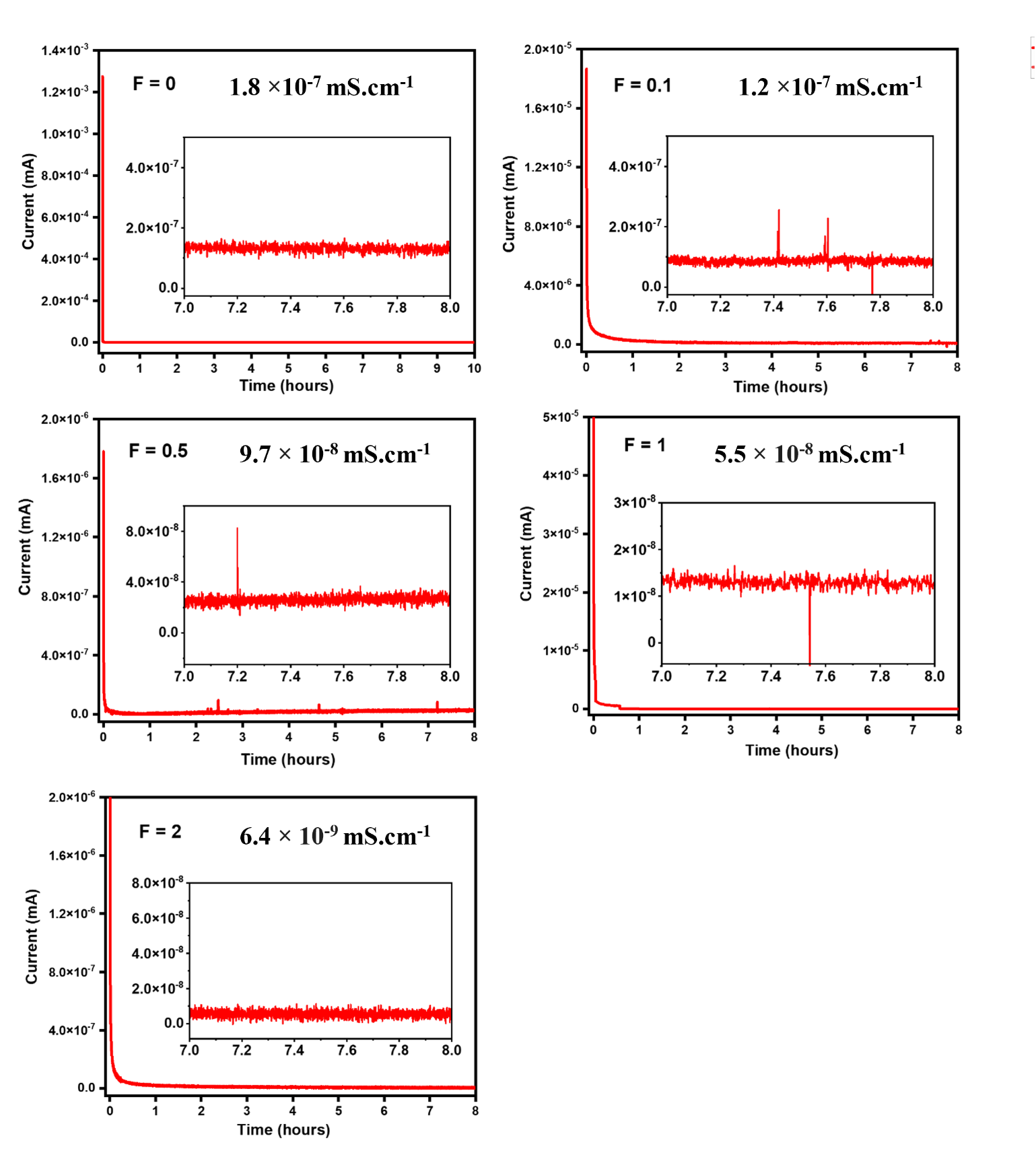
*Figure S5.** Electronic conductivity of different Li₂HfCl_6-x_F_x_ compositions measured by DC polarization. Chronoamperometry results for Ti/SE/Ti cells under a constant applied voltage of 0.5 V.


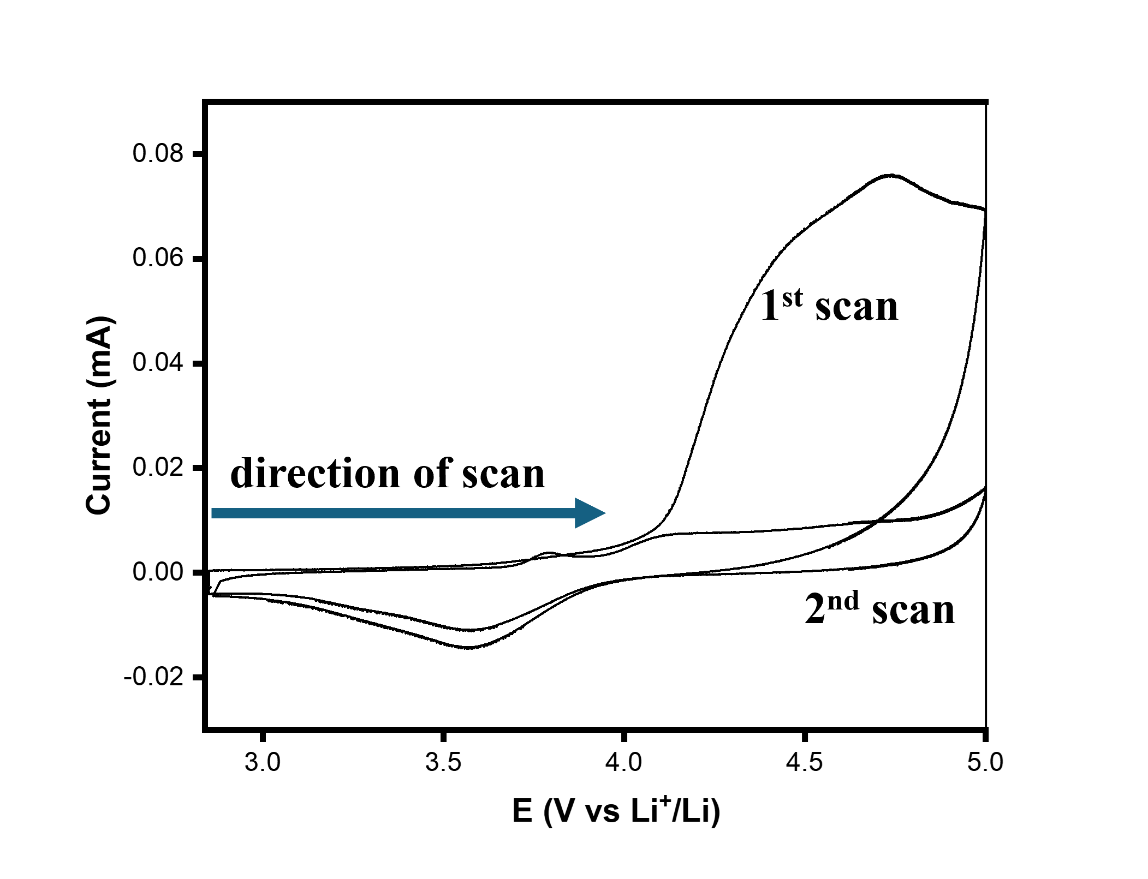


**Figure S6.** CV curves of the examined materials of the carbon-SE||In cells from OCV to 5.0 V followed by a reverse scan to 3 V and returning to OCV at 0.1 mV s^−1^. It is shown after the initial cycle, the oxidation peak nearly disappears, indicating the formation of a passivation layer at the interface between the carbon nanofiber and the SE.

**
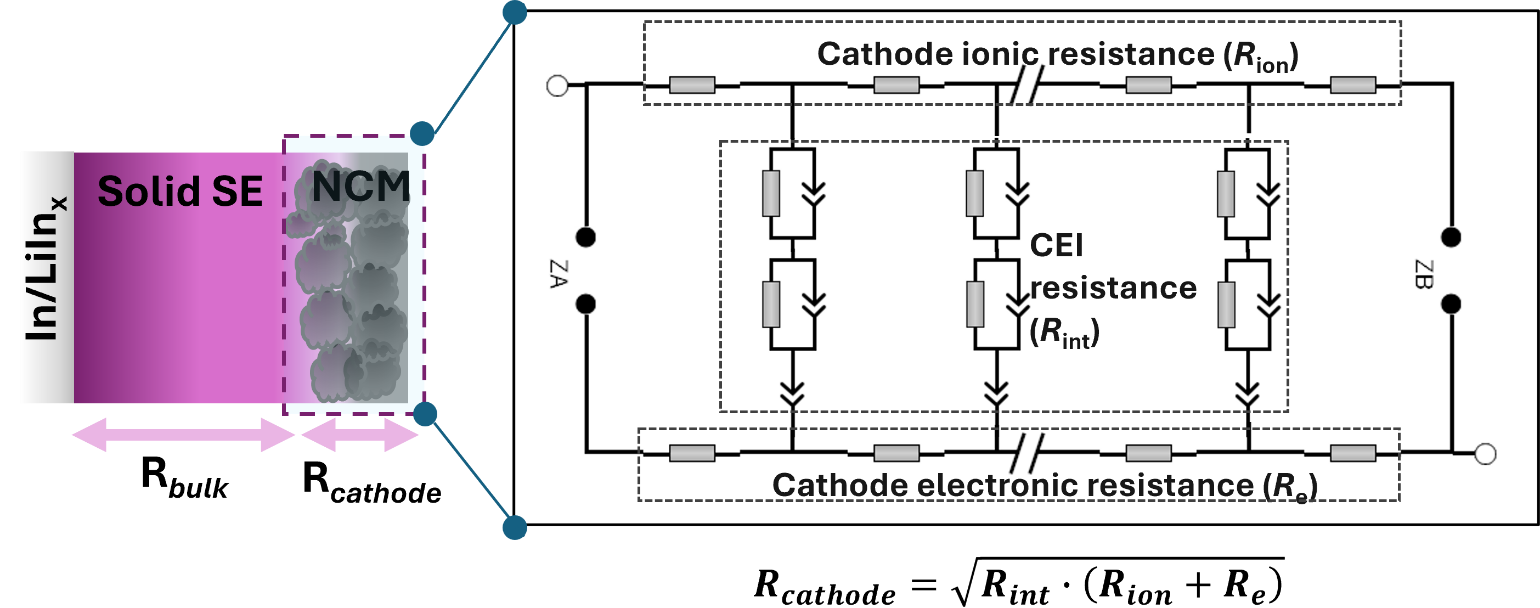
**

**Figure S7.** Transmission line model circuit used to fit the EIS results corresponding to interfaces in the battery.


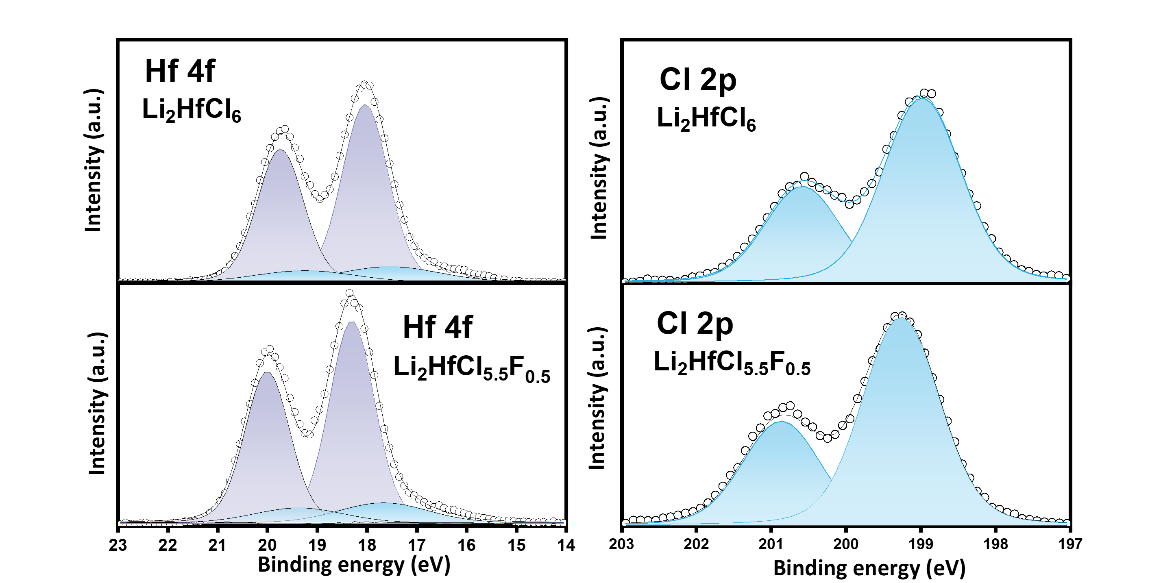


**Figure S8.** Comparison of XPS results (Cl 2p, and Hf 4f spectra) of pure Li_2_HfCl_6_ and Li_2_HfCl_5.5_F_0.5_. Cl 2p spectra show no discernable difference in spectral shape, although an increase in the binding energy (~0.5 eV) is observed in both the Cl 2p signals and Hf 4f signals for Li_2_HfCl_5.5_F_0.5_. A definitive assignment of the two components in the Hf 4f spectra is difficult due to the lack of reported binding energies for Hf-containing species in this region. However, we can tentatively assign the purple component at higher binding energy to Hf in the parent Li_2_HfCl_6_ and Li_2_HfCl_5.5_F_0.5_solid electrolytes, and the blue component (Hf 4f7/2 signal at ~ 17.6 eV) to HfO_x_ species arising from trace moisture contamination, based on XPS analysis of zirconium halides and oxides. Here, the binding energy of ZrCl_4_ (3d_5/2_at 183.7 eV) is higher in energy than ZrO_2_ (3d_5/2_at 182.3 eV), as reported in ref. 12 in the Supporting Information.

**
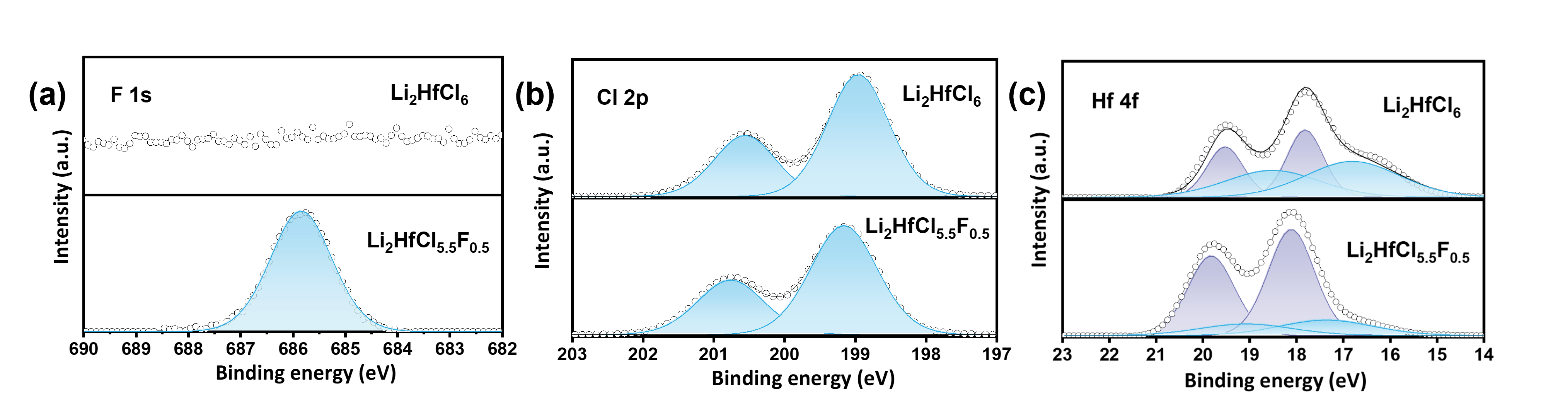
**

**Figure S9.** Comparison of XPS results of a cycled (between 2.8–4.3V vs Li^+^/Li) composite cathode for Li_2_HfCl_6_ and Li_2_HfCl_5.5_F_0.5_. XPS data are shown for (a) F 1s, (b) Cl 2p, and (c) Hf 4f spectra. The F 1s signal for Li_2_HfCl_5.5_F_0.5_ corresponds to that of LiF, but we cannot exclude potential contribution from Hf-F signals because of the large deviation in the reported LiF positions (685.5 ± 0.7 eV) and lack of report on Hf-F positions. Cl 2p spectra show no discernable difference in spectra shape.

We assign the purple component in the Hf 4f spectra in panel (c) to Hf in the parent Li_2_HfCl_6_ and Li_2_HfCl_5.5_F_0.5_ solid electrolytes, and the blue component to an interfacial reaction product with the NCM surface such as HfO_x_. Compared to the XPS signals of the pristine SEs in **Fig. S8**, the Hf 4f spectra exhibit clear changes upon cycling. A significant increase in the broad blue component in panel 4(c) (4f_7/2_ = 16.8 -17.3 eV) is observed for the cycled Li_2_HfCl_6_ composite, indicating the formation of HfO_x_ species originating from the interfacial reaction between CAM and the SE. In contrast, Li_2_HfCl_5.5_F_0.5_ shows minimal changes in that component compared with **Fig S8**, suggesting a lower extent of Hf oxidation. These XPS results indicate that fluorination in Li_2_HfCl_5.5_F_0.5_ effectively suppresses side reactions with NCM, consistent with the improved electrochemical performance observed in our battery data.

**
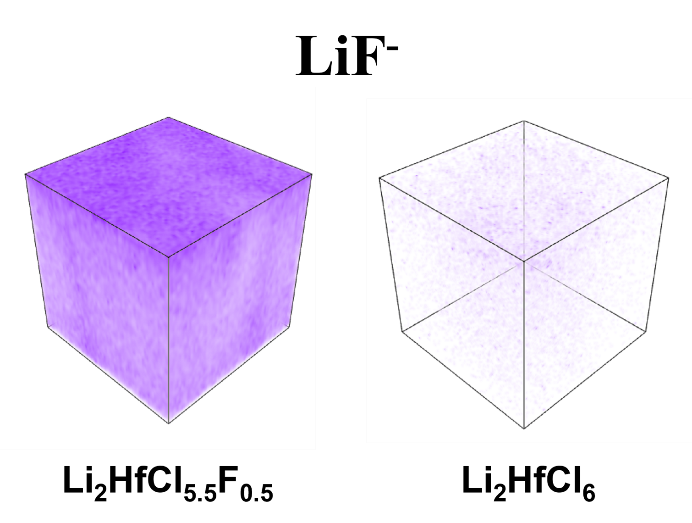
**

**Figure S10.** 3D reconstruction of LiF^-^ fragments obtained from ToF-SIMS results.

**List of Supplemental Tables**

**Table S1.** Atomic coordinates, occupation factor, and isotropic displacement parameters of Li_2_HfCl_6_ obtained from ToF neutron powder diffraction data collected at 300 K.

| *a* = 12.624(3) Å, *b* = 10.916(2) Å, *c* = 5.9309(2) Å; Space group: *Pnma* | | | | | | |
| --- | --- | --- | --- | --- | --- | --- |
| Atom | *x* | *y* | *z* | Occ. | *B*_iso_ | Multiplicity |
| Li | 0.891(5) | 0.576(8) | 0.477(10) | 0.27(3) | 0.2(2) | 8 |
| Li | 0.5992(18) | 0.408(2) | 0.565(3) | 0.73(3) | 0.2(2) | 8 |
| Hf | 0.375(2) | 0.25 | 0.002(3) | 0.595(13) | 0.81(19) | 4 |
| Hf | 0.379(2) | 0.25 | 0.479(4) | 0.405(13) | 0.1(2) | 4 |
| Cl | 0.8025(5) | 0.4092(4) | 0.7708(6) | 1 | 0.88(5) | 8 |
| Cl | 0.4623(5) | 0.75 | 0.3144(6) | 1 | 0.88(5) | 4 |
| Cl | 0.4512(4) | 0.4184(3) | 0.2406(7) | 1 | 0.88(5) | 8 |
| Cl | 0.2180(4) | 0.25 | 0.2277(11) | 1 | 0.88(5) | 4 |

**Table S2.** Atomic coordinates, occupation factor, and isotropic displacement parameters of Li_2_HfCl_5.5_F_0.5_ obtained from ToF neutron powder diffraction data collected at 300 K.

| *a* = 12.608(3) Å, *b* = 10.898(2) Å, *c* = 5.92222(9) Å; Space group: *Pnma* | | | | | | |
| --- | --- | --- | --- | --- | --- | --- |
| Atom | *x* | *y* | *z* | Occ. | *B*_iso_ | Multiplicity |
| Li | 0.898(4) | 0.566(6) | 0.481(7) | 0.50(5) | 2.0(11) | 8 |
| Li | 0.600(4) | 0.414(5) | 0.562(5) | 0.50(5) | 2.0(9) | 8 |
| Hf | 0.3858(17) | 0.25 | 0.012(4) | 0.553(15) | 1.0(2) | 4 |
| Hf | 0.377(3) | 0.25 | 0.483(5) | 0.447(15) | 1.0(3) | 4 |
| Cl | 0.80095 | 0.40899 | 0.76552 | 0.95 | 1.00(5) | 8 |
| Cl | 0.46193 | 0.75 | 0.30565 | 0.95 | 1.00(5) | 4 |
| Cl | 0.45169 | 0.41626 | 0.24197 | 0.95 | 1.00(5) | 8 |
| Cl | 0.21784 | 0.25 | 0.21948 | 0.95 | 1.00(5) | 4 |
| F | 0.80095 | 0.40899 | 0.76552 | 0.05 | 1.00(5) | 8 |
| F | 0.46193 | 0.75 | 0.30565 | 0.05 | 1.00(5) | 4 |
| F | 0.45169 | 0.41626 | 0.24197 | 0.05 | 1.00(5) | 8 |
| F | 0.21784 | 0.25 | 0.21948 | 0.05 | 1.00(5) | 4 |

**Table S3.** Quantitative structural data for Li_2_HfCl_6-x_F_+x_ (x = 0, 0.1, 0.5, 2) obtained by fitting the EXAFS data.

| Sample | Path | ACN ± 0.5 | σ^2^ (Å^2^)  ± 0.0002 | R (Å) ± 0.02 | ΔE_0_ (eV) | R fitting range | FT fitting range |
| --- | --- | --- | --- | --- | --- | --- | --- |
| Li_2_HfCl_6_ | Hf-Cl | 3.3 | 0.0020 | 2.28 | -2.9 | 1.28-3.99 | 1.78-12.38 |
|  | Hf-Cl | 2.7 | 0.0020 | 2.46 |  |  |  |
|  | Hf-Cl | 2.5 | 0.0020 | 2.27 | -5.0 | 0.95-4.01 | 1.64-11.72 |
| Li_2_HfCl_5.5_F_0.5_ | Hf-Cl | 2.5 | 0.0020 | 2.45 |  |  |  |
|  | Hf-F | 1.5 | 0.0032 | 2.0 |  |  |  |
|  | Hf-Cl | 2.1 | 0.0020 | 2.24 | -5.6 | 1.01-3.95 | 1.59-11.99 |
| Li_2_HfCl_4_F_2_ | Hf-Cl | 1.8 | 0.0020 | 2.42 |  |  |  |
|  | Hf-F | 2.3 | 0.0032 | 2.0 |  |  |  |

*ACN: average coordination number. σ^2^: Debye-Waller factor. R: bond length. ΔE: inner shell potential shift. R-factor for Li_2_HfCl_6_ (0.010); Li_2_HfCl_5.5_F_0.5_ (0.014); Li_2_HfCl_4_F_2_ (0.013).

**Table S4.** Interfacial reaction products and corresponding reaction energies for cathode/electrolytes derived from DFT calculations. The most energetically favorable reactions involving oxide chloride products are displayed.

|  |  | Reaction products | ΔE_rxt_ (eV per atom) |
| --- | --- | --- | --- |
| Li_0.25_NiO_2_ | Li_2_HfCl_6_ | NiCl_2_, LiClO_4_, LiCl, HfO_2_ | -0.173 |
|  | Li_2_HfCl_5.5_F_0.5_ | LiClO_4_, NiCl_2_, LiF, LiCl, HfO_2_ | -0.168 |
